# Using Intervention Mapping to Develop a Media Literacy-Based Smoking Prevention Program for Female Adolescents

**DOI:** 10.3390/ijerph18126305

**Published:** 2021-06-10

**Authors:** Sookyung Kim

**Affiliations:** College of Nursing, Yonsei University, Seoul 03722, Korea; sookyungkimm@gmail.com; Tel.: +82-2-2228-3365

**Keywords:** media literacy, smoking prevention, female, adolescent, web-based intervention, intervention mapping

## Abstract

Smoking prevalence among female adolescents in South Korea has increased gradually, despite a decreasing trend seen for male adolescents. Smoking scenes or cigarette advertisements in the media have influenced female adolescents’ initiation into smoking. It is therefore crucial to develop a smoking prevention program to enhance female adolescents’ smoking media literacy by implementing gender-specific approach. The purpose of this study is to describe how intervention mapping protocol (IMP) was used to develop a media literacy-based smoking prevention program (MLSP) for female adolescents. The IMP was used in six steps: needs assessment (literature review and focus groups comprising 24 female adolescents and 12 teachers), program goal setting, selection of intervention methods, production of program components and materials, program implementation planning, and program evaluation by ten experts and three adolescents. Six performance objectives and 14 change objectives were generated. Each module consisted of theory-based methods such as raising consciousness. Half of the modules covered topics regarding smoking media literacy, while half covered topics related to gender-specific intervention. The major advantages of utilizing IMP are that MLSP has been developed to reflect multiple perspectives, including of adolescents, teachers, and professors through a systematic process, and identified to be acceptable and valid.

## 1. Introduction

There is an increase in smoking prevalence among female adolescents in South Korea, compared to a decrease among male adolescents. The smoking rate of female adolescents decreased from 7.6% in 2009 to 2.7% in 2016, but then increased to 3.8% in 2019 [1]. Considering the tendency to underreport the smoking rate of women in Korea, the rate increase is predicted to be higher [2]. The daily smoking habits of female adolescents usually begin in the second year of middle school and the first year of high school (25%). Additionally, the smoking prevalence for females is highest among women in their 20s; some 9.7% of women in their 20s are smokers [3]. It is thus essential to prevent females from smoking in high school.

According to Primack and colleagues, smoking media literacy is defined as understanding, analyzing, appraising, and interpreting media messages about smoking [4]. Prior studies have found that adolescents smoking media literacy education had significant impact not only on quitting smoking, but also on preventing smoking [5,6]. It is known that adolescents tend to show a positive attitude toward smoking when they see cigarette advertisements as well as their peers’ smoking posts on social media [7]. In Korea, with the development of new media, the National Health Promotion Act, which regulates the promotion of various cigarettes such as e-cigarettes, was partially revised in 2020 [8]. Nevertheless, there are few legal restrictions on smoking scenes in social media (e.g., YouTube, Instagram) and web-based cartoons (hereinafter webtoons). Furthermore, female adolescents enjoy sharing more visually oriented platforms than male adolescents [9], and the sharing and viewing of selfies that show smoking behavior instills a positive image of smoking, which contributes to the sense that smoking is normal [10]. Therefore, it is crucial to improve the competency of smoking media literacy among female adolescents to prevent smoking.

On the other hand, tobacco companies have long emphasized the effects of smoking-induced sexuality and weight control [11,12], specifically targeting female populations with those advertisements [13]. Gender-specific marketing of cigarettes has long affected female adolescents, but most of the anti-smoking interventions have been gender-unresponsive [13]. The gender-specific approach is a method that recognizes gender norms and fully considers gender-specific needs [14], and is thus recommended for smoking prevention in women [13]. However, the evidence for gender-specific smoking prevention in female adolescents is still insufficient [15], and there are no studies that have attempted this approach in South Korea, despite the increasing demand for smoking prevention for female adolescents [16].

Among the theoretical frameworks explaining adolescents’ attempts and behaviors to smoke, the theory of planned behavior [17] is considered the most appropriate [18]. According to this theory, the smoking behavior of adolescents is determined by intention, and intention is determined by attitude toward behavior, subjective norm, and perceived behavioral control [17]. Smoking media literacy decreases positive attitudes toward smoking and promotes subjective norms that perceive social pressure not to smoke, thereby lowering smoking intentions and leading to changes in smoking behavior [4,19]. Self-efficacy, which is the same concept as perceived behavioral control [20], in controlling the smoking impulse even in the face of temptation, increases the predictive power of smoking initiation [21].

There is a need for a gender-specific smoking prevention program based on media literacy for female high school students to help them make healthy choices in an environment where various types of cigarettes and smoking behaviors appear. Intervention mapping, a systematic process, is useful for developing effective programs that can induce changes in health behavior [22]. Therefore, the purpose of this study is to describe how intervention mapping protocol (IMP) was used to develop a media literacy-based smoking prevention program (MLSP) for female high school students.

## 2. Materials and Methods

**Figure 1:**
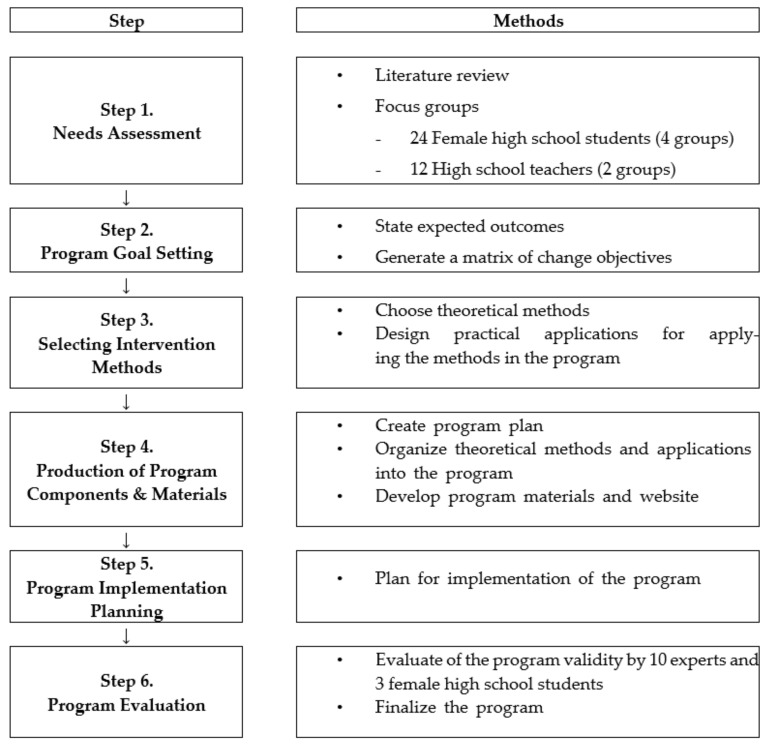
The process of program development.

The online-based program was developed using the IMP with the following six steps: (1) needs assessment, (2) program goal setting, (3) selection of intervention methods, (4) production of program components and materials, (5) program implementation planning, and (6) program evaluation by experts and adolescents (Figure 1).

### 2.1. Step 1—Needs Assessment

#### 2.1.1. Literature Review

We searched the PubMed, Embase, CHINAL, PsyInfo, and Cochrane databases for studies published in English between February 1990 and March 2020. The searching terms were ‘(teen* OR adolescen* OR child OR child* OR kid* OR youth* OR juvenile) AND (women OR woman OR female OR females OR girl OR girls OR gender) AND (smoking AND prevent* OR tobacco) AND prevent* AND ((media AND literacy OR health) AND literacy OR digital) AND literacy AND (intervention OR curriculum OR program OR education OR school)’. The criteria used to select studies for analysis included: (1) peer-reviewed articles, (2) published in English, and (3) experimental or quasi- experimental articles. The exclusion criteria were (1) research conducted not for adolescents, (2) for pregnant adolescents, and (3) research with a tobacco cessation program. We finally analyzed 10 out of 2423 studies after excluding articles that were not relevant to the topic.

#### 2.1.2. Focus Groups

The focus groups were with 24 female first-year high school students (four groups) and 12 female high school teachers (two groups) between July and August 2020. All participants were provided with information regarding the study purpose, and agreed to participate voluntarily. The student focus groups were conducted for 45 min at a female high school in Seoul. The teacher focus groups were conducted online using Zoom for 50–60 min. The focus groups were carried out by researchers trained in moderating focus groups.

Before each focus group, participants were informed of the research purpose, the process, including that the focus groups would be recorded, and that the data would be anonymous. The semi-structured focus groups were conducted through questions, including female students’ reasons for smoking, experiences of smoking scenes and cigarette advertisements in various media, and their preferred content and methods of smoking prevention programs. All verbal data were recorded and transcribed, and content analysis [23] was performed. A researcher repeatedly read the transcribed data and conducted open-coding of meaningful units. Then, similar semantic phrases were grouped by constants, facilitating the reclassification and restructuring of the codes to identify subthemes and themes. The study protocol was approved by the Institutional Review Board of the corresponding author’s institution (IRB No. Y-2020-0078).

### 2.2. Step 2—Program Goal Setting

The second step is to lay the foundation of the program by specifying what needs to be changed as a result of the intervention, deriving “the matrix of change objectives”. By applying an MLSP for female high school students, the expected result to be finally reached is stated as “reducing smoking intention.” The performance objectives were generated based on the core principle of media literacy education in the United States [24] and the results of the focus groups of needs assessment.

### 2.3. Step 3—Selection of Intervention Methods

The third step involves selecting theoretical methods and practical applications to achieve each change objective, all of which are divided into attitudes, subjective norm, and self-efficacy, determinants of the theory of planned behavior (TPB).

### 2.4. Step 4—Production of Program Components and Materials

The fourth step is to make the overall draft MLSP, including goal, contents, teaching methods, theoretical methods, time, and type of online methods based on the results of the prior steps. The draft of the MLSP contents and composition was decided in accordance with attitudes, subjective norm, and self-efficacy of the TPB, so that female adolescents can make healthy choices related to smoking. When constructing program elements, literature related to smoking, other than the literature review, was evidenced.

### 2.5. Step 5—Program Implementation Planning

The fifth step is to establish a plan to apply and implement the developed MLSP among first-year female high school students. Researchers visited and had discussions with teachers in charge of smoking prevention programs at female high schools. In addition, in order to specifically establish an implementation plan applicable to students, we discussed the school’s current teaching method in the COVID-19 situation, and the feasibility of applying online programs.

### 2.6. Step 6—Program Evaluation

To verify the validity of the expert group, eight nursing professors, with expertise in smoking-related research and development of intervention programs, together with two high school health teachers with more than 20 years of experience were selected. The program evaluation survey was sent via email, consisting of a 4-point Likert scale for the appropriateness of the four items (title, goal, content, and method), for the components of the program (1 = “not relevant”, 2 = “somewhat relevant”, 3 = “quite relevant”, 4 = “highly relevant”), and opinions could be written separately. According to Lynn’s method [25], I-CVI was calculated by dividing the number of people indicated on three or four points out of the 4-point scale by the total number of people, and reviewing and correcting the items with I-CVI less than 0.78 [26]. The applicability of the program content and usability of the web interface were validated by three female adolescents.

## 3. Results

### 3.1. Step 1—Needs Assessment

#### 3.1.1. Literature Review

In the first stage of needs assessment, the MLSP was planned as pre-post design with a control group, and the number of program modules was set at eight modules for an intervention period of four weeks based on the 10 results of the literature review. A web-based method was chosen due to its easy accessibility for students, and the fact that it can be replicated and used in the future [27,28].

#### 3.1.2. Focus Groups

In the focus groups of the second stage of needs assessment, the content and methods that should be included in the MLSP were derived. Female adolescents and teachers perceived that the reasons for smoking among female adolescents are the influence of peer relationships and pursuit of a “cool” look. Therefore, the program should include content regarding the influence of peer relationships. It was confirmed that there was a gap between teachers and adolescents regarding the awareness of the experience and influence of smoking on social media platforms such as Instagram and Twitch. Only a few young teachers speculated on adolescents’ smoking-related activities on social media, and many teachers were unaware that students upload and communicate smoking-related photos or videos on social media. While most of the teachers agreed that smoking scenes and cigarette advertisements in traditional media such as TV and movies are important factors influencing adolescents’ smoking, many teachers were not aware of the effect social media has. Not only are adolescents exposed to smoking media content, but they also produce such content when they upload their own smoking photos or posts on their social media accounts, or when they share this type of content with each other. The “cool” look of a smoking female should therefore be addressed in order to reduce its influence on female high school students who wish to imitate such a look. Other issues to be addressed are enhancement of adolescents’ inner strength, the effect of smoking on themselves and their family, peers (e.g., changes in a look), and the impact of e-cigarettes on health. It was also mentioned that too much focus on the impact of smoking on pregnancy and childbirth should be avoided. Participants recommended that educational methods utilizing participatory activities, such as discussion and experience, should be adopted.

### 3.2. Step 2—Program Goal Setting

During the second step, six performance objectives were selected after the needs assessment to determine the effects of the program on the smoking intention of female adolescents. Fourteen change objectives were then selected as determinants of attitude, subjective norm, and self-efficacy (Table 1).

### 3.3. Step 3—Selection of Intervention Methods

In the third step of selecting intervention methods, appropriate theoretical methods were determined according to the change objectives for attitude, subjective norm, and self-efficacy. The practical application methods were then connected (Table 2). For example, to meet the subjective norm 6 of change objectives (recognize that most adolescent feel it is important to know that smoking during adolescence won’t be a nice look in the long run), the “self-reevaluation” theoretical method was selected, and the approach of virtually experiencing skin aging caused by smoking through an app was then configured to be applied [29].

### 3.4. Step 4—Production of Program Components and Materials

In the fourth step of producing program components and materials, the MLSP comprises eight modules for a duration of four weeks. The program is conducted twice a week and is provided once a week with real-time participation on Zoom, and website participation on Padlet. Designed in an adolescent-friendly way for participants who regularly use social media, the Padlet website is similar to social media, letting users check their friends’ comments and communicate with clicking ♡. Padlet was utilized for self-directed intervention, and modules in Padlet consistently consisted of the following format: Attendance, Activity 1—watching the short video for an explanation of today’s module made by the researcher, Activity 2—uploading material made by themselves such as posters, Activity 3—commenting on the postings by friends, New things for process evaluation, and taking notice of the next module date and time. An example of a Padlet module, in which the contents were translated from Korean to English, can be found at https://padlet.com/sookyungkimm/cpvsq0rwhdy2r68e (accessed on 27 May 2021). Four modules (M 2, 4, 7, and 8) consisted of topic related to the smoking media literacy. The contents consisted of (M2) finding inappropriate smoking scenes and ads in daily life; (M4) analyzing smoking scenes and advertisements; (M7) increasing the sensitivity of smoking media literacy; and (M8) making smoking prevention videos for the public, including friends, where the teaching methods were organized discussions, activities, and video making. Another four modules (M1, 3, 5, and 6) consisted of gender-specific smoking prevention topics, which were identified in the literature review and focus groups. The contents consisted of (M1) changes in the body caused by smoking, (M3) the culture of respecting smoking refusal, (M5) realizing the reality of e-cigarettes, and (M6) the impact of smoking on future goals, in which the teaching methods were organized as virtual experiences, discussions, and poster making. The guidelines for each module were developed based on objectives, content, activity, and evaluation.

### 3.5. Step 5—Program Implementation Planning

In the fifth step, the researcher discussed program implementation planning for students with the teacher. The delivery method of the MLSP, which planned the entire online approach, was confirmed to be appropriate in accordance with the school situation where the entire classes had been provided online due to COVID-19. The researcher tried to effectively plan in a real-time module using Zoom, which, by increasing communication with peers participating online would, according to a teacher’s suggestion, improve the continuity of participation in the program. The MLSP will be provided as part of additional school activities that allow students to participate in the program to prevent smoking. Participants will be recruited from first-year female high school students who have no smoking experience, or who have quit smoking for more than six months. The implementer of the MLSP will be a researcher with experience teaching students general smoking prevention programs in school. The program implementer will provide an online link to participants for self-directed modules every Wednesday, and interact with participants on Zoom every Sunday. To control the quality of the intervention, anonymous comments will be received from participants once a week. In Module 8, the group campaign video will be uploaded to the Padlet website, where all participants will watch each group’s video, and leave comments. After all modules are finished, the researcher and participants will share their feelings and opinions about participation in the MLSP, including discussion of the group campaign video at school. To ensure effective participation, it is important to sufficiently explain the purpose of the program to students, explain what they should do, and to emphasize interesting intervention methods such as games, rather than boring lectures. While the module is in progress, it is necessary to praise students for their good work, express interest, and provide many opportunities for online discussions and group work.

### 3.6. Step 6—Program Evaluation

In the sixth step, the initial program had a content validity index of 0.97 by 10 experts. The item-specific validity index was 0.96 for the title, 0.95 for the objectives, 0.98 for the contents, and 0.99 for the methods. The program was finalized by changing the order of modules in order to increase connection by continuously educating on media literacy-related topics, and to ensure that feedback on web-based modules should be linked in real-time modules. According to the suggestion of an expert, female-specific contents such as the risk of birth defects and breast cancer caused by smoking were added to Module 1. The topics were modified to clearly reveal the meaning of the modules. Three first-year female high school students evaluated that it was easy to use real-time methods through Zoom, but on the Padlet website, it was mentioned that if the information about the activity was simple and clear, it would be more understandable. Directions on Padlet were hence modified according to the students’ evaluations.

The final MLSP for female high school students is presented Table 3. Modules 2, 3, 7, and 8 cover topics regarding exploring and analyzing smoking scenes or cigarette advertisements, and creating media content related to smoking. Modules 1, 4, 5, and 6 cover topics such as recognizing the harmful effects of smoking on female adolescents, understanding the reality of e-cigarette use, forming a school climate in which a friend’s refusal of smoking is respected, and realizing the impact of smoking on their future.

## 4. Discussion

The program developed in this study is useful as it is an online smoking prevention program with a gender-specific approach focusing on smoking media literacy, that fully considers the environment in which the media and its influence have increased. In terms of program content, the existing media literacy smoking prevention programs mainly addressed television [30], smoking scenes in movies and mass media [5], and cigarette advertisements. However, this study is differentiated in that it includes social media (i.e., YouTube, Instagram, etc.) and webtoons which have recently gained influence.

In terms of programming methods, the existing web-based media literacy smoking prevention programs include learning to play alone [28], games to play alone [31], and activities involving communication with online avatars [27,32], but it was confirmed that there were relatively few interventions which included online interactive communication. This study confirmed the needs for active methods and discussions of female high school students in the program development stage. In addition, the National Association for Media Literacy Education recommended that an interactive and activity-oriented method should be applied to facilitate interactions and promote media literacy [24]. Therefore, although it was an online program, it was developed to be interactive and activity-oriented. The core intervention development for adolescents is to be able to draw their attention to have an interest in the program, and actively participate in order to achieve the learning goals [33]. With this, the program was meaningful, as it was developed to be an engaging and fun method of communicating with educators.

In the program development process, there were constructed points that aimed to be gender-specific. According to a previous study, female adolescents have increased motivation to start smoking in order to get along with or to hang out with their peers [34]. Based on the results of the needs assessment in the present study, using the smoking refusal skills, it can be seen that the group discussion was devised to “encourage a social atmosphere that respect friends’ refusal” and that the gender-specific content was remarkable. In addition, in the case of female adolescents, when their stress level is higher than that of male adolescents [35] or when they have low self-esteem [36], they have a higher tendency to smoke. Reflecting the result that it is necessary to prevent smoking by increasing self-esteem by means of setting life goals and improving internal strength, Module 6 is focused on their dreams and how smoking will affect their life goals. The participation experience confirmed that the learning goal was achieved when students expressed that they would not smoke as it may threaten their dreams. After gathering expert feasibility opinions, the gender-specific content additions were the increased risk of female cancer due to smoking [37] and the appearance of the fetus during maternal smoking during pregnancy. In the Korean social context, where marriage and childbirth are options, studies have shown that there is antipathy for linking smoking to pregnancy in female smokers, thus it is important to be careful when linking female smoking and maternal aspects [38]. Focusing on the current health of female adolescents, the aforementioned contents were carefully reviewed to avoid difficulties in future due to ignorance.

This program is based on the theory of planned behavior designed to lower smoking intentions through cognitive changes regarding smoking in female adolescents. After setting program goals according to the process of intervention mapping [22], the program was organized according to a systematic process. In a previous study developed according to the intervention mapping process for gender-specific smoking cessation interventions for women in Brazil [39], women, community health workers, healthcare workers, and administrators took part in the needs assessment process. Although there were difficulties in the process, sustainable interventions were developed by investigating specific needs for various stakeholders. In this study, the program was also developed with the participation of female adolescents, teachers, and professors. However, further research to develop a multidimensional smoking prevention program to include extended stakeholders (i.e., public health center administrators, policy makers, etc.) should be considered in the future. Another limitation of this study is that intervention mapping was developed only at the personal level by focusing on changes to individual socio-cognitive factors. It is necessary to develop the MLSP to be multi-level, such as school and community levels, for an integrative program.

## 5. Conclusions

It is important that an online smoking prevention program developed for female high school students with a focus on media literacy is developed, given the environment wherein the media and its influence has become a part of their daily lives. The MLSP was found to be valid and acceptable for female adolescents, and it is expected that this program could be utilized at school levels to reduce the smoking intention of female adolescents. Further study is needed to evaluate the effects of this intervention.

## Figures and Tables

**Table 1:** The matrix of change objectives.

Expected Outcome: Female High School Students Decrease Their Smoking Intention			
Performance Objective	Personal Determinant		
	Attitude	Subjective Norm	Self-Efficacy
1. Identify the facts that receive and produce media influence related to smoking for adolescent	A1. Recognize media messages related to smoking contain embedded values		SE1. Express confidence to explore smoking scenes or cigarettes ads which influence adolescents in media
2. Raise the sensitivity of smoking scenes or cigarettes ads in media		SN2. Encourage the social influences of unacceptable smoking in media, when others are numb to smoking scenes or cigarettes in media	SE2. Express confidence to analyze smoking scenes or cigarette ads skeptically
3. Advocate for smoking prevention to public	A3. Express taking responsibility for their own media use	SN3. Recognize that most adolescents feel it is important to create heathy social media contents	SE3. Express confidence in one’s ability to use their skills to construct media related to smoking by their own meanings
4. Determine life goals to improve self-esteem			SE4. Express confidence about setting life goals
5. Apply healthy peer relationship by forming naturally refusal culture when suggesting smoking	A5. Express positive feelings about friends’ refusal to smoke	SN5. Encourage a social atmosphere that respect friends’ refusal	SE5. Express confidence about refusing smoking suggestion
6. Apply relevant knowledge of smoking effects for adolescent	A6. Express negative feelings about weight loss and skin aging effects of smoking	SN6. Recognize that most adolescent feel it is important to know that smoking during adolescence won’t be a nice look in the long run	SE6. Express confidence in one’s ability to use knowledge about e-cigarettes to make a decision when peers nudge to smoke

A = Attitude; SE = Self-efficacy; SN = Subjective norm.

**Table 2:** Theoretical methods and practical applications.

Determinant	Change Objective	Theoretical Method (Theory)-Definition	Practical Application
Attitude	A6	Consciousness raising (HBM)-providing information, feedback, or confrontation about the causes, consequences, and alternatives for a problem or a problem behavior	Lecture Discussion
	A1	Active learning (SCT)-encouraging learning from goal-driven and activity-based experience	Video Searching and posting activity Commenting on each other
	A3	Media advocacy (Models of Community Organization)-expose environmental agents’ behaviors in the mass media to order to get them to improve health-related conditions	Creating video Commenting on each other
	A5	Shifting perspective (Theories of Stigma and Discrimination)-encouraging taking the perspective of the other	Lecture Group discussion
Subjective norm	SN6	Self-reevaluation (TTM)-encouraging combining both cognitive and affective assessment of ones’ self-image with and without an unhealthy behavior	Virtual photoaging activity
	SN5	Mobilizing social support (Diffusion of Innovations Theory)-prompting communication about behavior change in order to provide instrumental and emotional social support	Group discussion
	SN2	Framing (Protection Motivation Theory)-using gain-framed messages emphasizing the advantages of performing the healthy behavior	Lecture Discussion
	SN3	Media advocacy (Models of Community Organization)-expose environmental agents’ behaviors in the mass media to order to get them to improve health-related conditions	Creating video as group activity Commenting on each other
Self-efficacy	SE1	Active learning (SCT)	Searching and posting activity
	SE2	Active learning (SCT)	Sharing opinion Lecture Real time quiz
	SE5	Resistance to social pressure (TPB)-Stimulating building skills for resistance to social pressure	Video Sharing opinions
	SE4	Goal setting (Goal Setting Theory)-Prompting planning what the person will do, including a definition of goal-directed behaviors that result in the target behavior	Video Writing about life goals and the impact that smoking will have on them
	SE2	Active learning (SCT)	Lecture Discussion
	SE6	Consciousness raising (HBM)	Making a poster

A = Attitude; SE = Self-efficacy; SN = Subjective norm; HBM = Health belief model; SCT = Social cognitive theory; TPB = Theory of planned behavior; TTM = Transtheoretical model.

**Table 3:** Final online media literacy-based smoking prevention program for female adolescents.

Module & Topic					
Goal	Contents	Teaching Method	Theoretical Method	Time (min)	Online Method
**Module 1. The physically harmful effects of smoking**					
SN6. Recognize that most adolescents feel it is important to know that smoking during adolescence won’t be a nice look in the long run A6. Express negative feelings about physical change including aging effects of smoking	·Experience virtual photoaging (using “Smokerface App”) ·Reality of physical changes of smoking (possible risks of breast cancer, facial changes and birth defects)	·Virtual photoaging activity ·Discussion ·Lecture	·Consciousness raising ·Self-reevaluation	40	Real time (Zoom)
**Module 2. Finding Inappropriate Smoking Scenes and Ads in Daily Life**					
SE1. Express confidence to explore smoking scenes or cigarettes ads which influence adolescents in media A1. Recognize media messages of smoking scenes or cigarettes ads	·Searching for smoking scenes or cigarettes ads in various media ·Meaning of the media messages and embedded values	·Video ·Searching and posting activity ·Commenting on each other	·Active learning	20	Website (Padlet)
**Module 3. Analyzing Inappropriate Smoking Scenes and Ads**					
SE2. Express confidence to analyze smoking scenes or cigarettes ads skeptically	·Expressing own ideas about smoking scenes or ads through multiple types of media (e.g., ads on website, Instagram, Webtoon, drama)	·Sharing opinions ·Lecture ·Real-time quiz	·Framing ·Active learning	40	Website (Zoom)
**Module 4. The reality of e-cigarettes?**					
SE6. Express confidence in one’s ability to use knowledge about e-cigarettes to make decision when peers nudge to smoke	·Making a poster to let your friends realize the reality of e-cigarettes	·Video ·Making a poster ·Commenting on each other	·Consciousness raising	40	Real time (Padlet)
**Module 5. The culture of respecting smoking refusal**					
A5. Express positive feelings about friends’ refusal to smoke SN5. Encourage a social atmosphere that respect friends’ refusal	·Forming school rules for an atmosphere respecting friends’ refusal to smoke	·Lecture ·Group discussion	·Information about others’ approval ·Mobilizing social support ·Shifting perspective	40	Real time (Zoom)
**Module 6. The obstacle to my goals is smoking**					
SE4. Express confidence about setting life goals SE5. Express confidence about refusing smoking suggestion	·Thinking about and making life goals ·Thinking about relationship between smoking and life goals	·Video ·Writing about your life goals and the impacts that smoking will have on them	·Resistance to social pressure ·Goal setting	20	Website (Padlet)
**Module 7. Increasing the sensitivity of smoking media literacy**					
SE2. Express confidence to analyze smoking scenes or cigarette ads skeptically SN2. Encourage the social influences of unacceptable smoking in media, when others are numb to smoking scenes or cigarettes in media	·Analyzing and appreciating the media related to smoking scenes and cigarettes ads	·Lecture ·Discussion	·Framing ·Active learning	40	Real time (Zoom)
**Module 8. Making video for smoking prevention**					
SE3. Express confidence in one’s ability to use their skills to construct media related to smoking by their own meanings SN3. Recognize that most adolescents feel it is important to create heathy social media contents	·Making videos for raising awareness about smoking prevention	·Creating video as group activity ·Commenting on each other	·Media advocacy	20	Website (Padlet)

A = Attitude, SE = Self-efficacy, SN = Subjective norm.

## Data Availability

The data presented in this study are available on request from the corresponding author. The data are not publicly available to protect confidentiality of the research participants.
